# Barriers Prevent Patient Access to Personalized Therapies Identified by Molecular Tumor Profiling of Gynecologic Malignancies

**DOI:** 10.3390/jpm5020165

**Published:** 2015-05-21

**Authors:** R. Tyler Hillman, Kristy Ward, Cheryl Saenz, Michael McHale, Steven Plaxe

**Affiliations:** 1 Rebecca and John Moores UCSD Cancer Center, Department of Reproductive Medicine, Division of Gynecologic Oncology, La Jolla, CA 92093, USA; E-Mails: csaenz@ucsd.edu (C.S.); mtmchale@ucsd.edu (M.M.); splaxe@ucsd.edu (S.P.); 2 Division of Gynecologic Oncology, College of Medicine-Jacksonville, University of Florida, Jacksonville, FL 32209, USA; E-Mail: kristy.k.ward@gmail.com

**Keywords:** targeted therapies, tumor markers, genetics, molecular biology, next generation sequencing

## Abstract

*Objective.* This study was designed to evaluate the ability of commercial molecular tumor profiling to discover actionable mutations and to identify barriers that might prevent patient access to personalized therapies. *Methods*. We conducted an IRB-approved retrospective review of 26 patients with gynecologic malignancies who underwent commercial tumor profiling at our institution during the first 18 months of test availability. Tumor profiles reported targeted therapies and clinical trials matched to patient-specific mutations. Data analysis consisted of descriptive statistics. *Results*. Most patients who underwent tumor profiling had serous epithelial ovarian, primary peritoneal, or fallopian tube carcinoma (46%). Patients underwent profiling after undergoing a median of two systemic therapies (range 0 to 13). A median of one targeted therapy was suggested per patient profile. Tumor profiling identified no clinically actionable mutations for seven patients (27%). Six patients sought insurance approval for a targeted therapy and two were declined (33%). One patient (4%) received a targeted therapy and this was discontinued due to tumor progression. *Conclusions*. There are formidable barriers to targeted therapy for patients with gynecologic malignancies. These barriers include a dearth of FDA-approved targeted agents for gynecologic malignancies, lack of third party insurance coverage and limited geographic availability of clinical trials.

## 1. Introduction

“Personalized medicine” is a popular term that refers to prognostic or therapeutic decisions that are made by analyzing patient specific molecular biomarkers. When the term is applied to cancer treatment, “personalized medicine” is best understood as the culmination of a historical trend towards an ever finer classification of tumor biology [1]. Before the development of molecular techniques, the classification of solid tumors was primarily based on site of origin and microscopic histological appearance. The development of immunohistochemical staining and fluorescence *in situ* hybridization (FISH) technology revolutionized the study of tumor biology, and both techniques have allowed for the sub-classification of tumors based on the presence of molecular biomarkers. Biomarker-based classification may provide prognostic information and may also influence the selection of adjuvant therapy, such as the decision to prescribe an aromatase inhibitor to a patient undergoing treatment for estrogen-receptor positive breast cancer [2].

Non-small cell lung cancer (NSCLC) has emerged as a disease in which personalized tumor biomarker detection has already become the standard of care. In NSCLC, separate testing for EGFR mutations and for ALK re-arrangement status predict responsiveness to families of kinase inhibitors that target these molecules [3]. Treatment of these tumors is therefore guided by the identification of patient-specific biomarkers. Despite recent large scale efforts to map the genomic landscape of gynecologic malignancies [4,5], until 2014 there were no FDA-approved predictive biomarkers that could be used to guide therapy choice in patients with these tumors. FDA approval of olaparib in December 2014 for use in ovarian cancer patients with germ line BRCA mutations who have received multiple prior chemotherapies represents a first step towards the clinical use of molecular biomarkers in therapy selection for gynecologic malignancies [6]. The development of new laboratory techniques for identifying and screening targeted therapies is accelerating [7] and although the future will likely bring FDA approval of additional targeted therapies, today very few patients with gynecologic malignancies are candidates for olaparib. The ever-decreasing cost of high throughput genetic sequencing has now allowed this technology to be applied in clinical practice, holding forth the promise that additional patient-specific therapeutic biomarkers may soon be identified for use in gynecologic malignancies [1].

One approach to personalized medicine in the treatment of gynecologic malignancies has been to identify patient-specific oncogene mutations using high throughput sequencing, then cross-reference these mutations with FDA-approved targeted drugs to identify therapies with theoretical efficacy. For example, the identification of an activating EGFR mutation in tissue taken from a patient sample of endometrial adenocarcinoma might suggest the theoretical utility of a kinase inhibitor such as erlotinib. A biomarker-based approach to targeted cancer therapy will soon be tested prospectively under the NCI-MATCH trial schema [8]. In this planned series of phase II clinical trials, patients with progressive solid tumors or lymphoma will undergo tumor sequencing followed by treatment with targeted therapies based upon patient-specific mutation patterns, with the primary endpoint of tumor response. While awaiting the results of NCI-MATCH trials, the decreasing cost of high throughput sequencing and increasing demand for “personalized medicine” has led to the development of commercial tumor profiling services.

The commercial application of next-generation sequencing to biomarker identification in solid tumors reached widespread availability in 2013. In the absence of prospective data, proper patient selection and overall utility of such testing remains unknown. To better understand the clinical application of biomarkers identified using next generation sequencing, we conducted a retrospective review of 26 sequential patients with gynecologic malignancies who underwent commercial molecular tumor profiling at our institution during the first 18 months of test availability.

## 2. Results

Twenty-six patients with gynecologic malignancies underwent tumor profiling at our institution between the initial availability of testing in 2013 and April of 2014 (Table 1). The median age at diagnosis was 55 years (range 16 to 75 years). Of those patients who underwent tumor profiling, 12 patients had serous epithelial ovarian, primary peritoneal, or fallopian tube carcinoma (46.1%), four patients had uterine leiomyosarcoma (15.3%), three patients had endometrial adenocarcinoma (11.5%), and two patients had cervical adenocarcinoma (7.6%). Of the remaining five patients, one was diagnosed with small cell neuroendocrine carcinoma of the cervix, one with apocrine adenocarcinoma of the vulva, one with ovarian granulosa cell tumor, one with clear cell carcinoma of the cervix, and one with uterine carcinosarcoma.

**Table 1:** Summary of molecular profiling results by tumor type. The primary results of molecular profiling performed on samples from the 26 patients in this series are summarized in this table.

	Serous Ovarian, Fallopian Tube, and Primary Peritoneal (N = 12)	Endometrial Adenocarcinoma (N = 3)	Uterine Leiomyosarcoma (N = 4)	Other (N = 7)	Overall (N = 26)
Median Age at Diagnosis (years)	58	64	50	43	55
Median Time From Diagnosis to Performance of Tumor Profiling (months)	47.5	15.0	65.0	37.0	42.0
Median Cytotoxic Chemotherapies Received Prior to Collection of Sample for Tumor Profiling	3	1	4	1	2
% of Profile Samples Taken From Primary Tumor (*vs.* recurrence)	41.6% (5/12)	66.6% (2/3)	25% (1/4)	42.8% (3/7)	42.3% (11/26)
Median # of Genetic Alterations Identified per Tumor Profile [range]	3 [1 to 6]	10 [8 to 11]	2 [2 to 3]	3 [1 to 5]	3 [1 to 11]
Median # of Potential Targeted Therapies Identified per Tumor Profile [range]	1 [0 to 6]	3 [1 to 6]	1 [0 to 4]	1 [0 to 2]	1 [0 to 6]
% of Tumor Profiles Identifying No Potential Targeted Therapy	25.0% (3/12)	0.0% (0/3)	50.0% (2/4)	28.5% (2/7)	26.9% (7/26)
Median # of Clinical Trials Identified per Tumor Profile [range]	6 [2 to 13]	8 [6 to 12]	2 [0 to 4]	4 [0 to 9]	4 [0 to 13]

The majority of patients received at least one course of cytotoxic chemotherapy prior to the collection of tissue for tumor profiling (24 out of 26, 92.3%). Of the two patients who had received no cytotoxic therapy prior to the collection of tissue for tumor profiling, one was diagnosed with leiomyosarcoma while the other was diagnosed with recurrent endometrial adenocarcinoma and had not received adjuvant therapy following her initial surgery. Following the diagnosis of recurrent disease, this latter patient received intensity modulated external beam radiation to the pelvis, which was administered before tissue was obtained for tumor profiling. Three additional patients received pelvic external beam radiation before tissue was obtained for tumor profiling.

Ten of the 26 patients underwent tumor profiling without having first entered disease remission and among this group testing was performed at a median of 7.5 months after pathologic diagnosis (range from 2 to 52 months). The remaining 16 patients had tumor profiling performed while undergoing treatment for recurrent disease. Among these patients, tumor profiling was performed at a median of 57 months after initial pathologic diagnosis (range from18 to 180 months) and a median of 24 months after the diagnosis of recurrent disease (range from 1 to 93 months). Of these 16 patients with recurrent disease, 8 had tumor profiling performed on tissue from their primary tumor and 8 had tumor profiling performed on tissue from a recurrent disease site.

**Figure 1:**
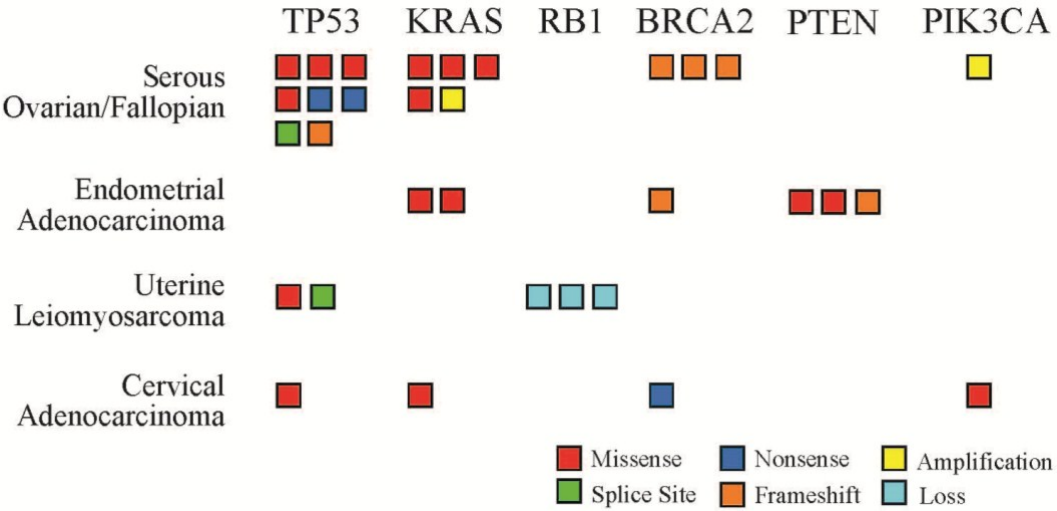
Number and type of genetic alterations identified by tumor profiling. Six genes were mutated in multiple patient samples across the four most common tumor types in this series. Each box represents one patient sample. Mutation types include missense (red), splice site (green), nonsense (dark blue), frame shift (orange), amplification (yellow), and gene loss (light blue).

FoundationOne^TM^ (Foundation Medicine, Cambridge, MA, USA) tumor profiling identified a median of 3 genomic alterations per tumor sample (range 1 to 11 alterations). Significantly more genomic alterations were identified in tumor samples from endometrial adenocarcinomas (mean 10, 95% CI [8.27, 11.73]) than for all other tumor types (mean 3, 95% CI [2.43, 3.31]) (two-sample *t*-test, *p* < 0.0001). These genomic alterations included missense mutations, nonsense mutations, frame shifts, gene amplifications and gene deletions. The frequency and type of genomic alteration varied by tumor histology and gene locus (Figure 1). TP53 was the most frequently mutated gene locus with alterations noted in 46.1% of tumor samples (12 of 26), followed by KRAS mutations (9 of 26, 34.6%) and BRCA2 mutations (6 of 26, 23.0%).

The FoundationOne^TM^ tumor profile report suggests relevant clinical trials and potential targeted therapies based on the mutation pattern identified in the tumor sample. Of the targeted therapies identified by tumor profiling among these 26 patients, none are FDA approved for use in each individual patient’s tumor type or for any gynecologic malignancy. For each patient sample, tumor profiling identified a median of one therapy with FDA approval for use in at least one non-gynecologic tumor type (range 0 to 6 therapies with potential benefit identified per patient profile). In addition, a median of four relevant clinical trials was identified per patient sample (range 0 to 13 clinical trials). No FDA approved therapy with potential benefit was identified for 27% of patients (7 of 26) and two of these patients also had no relevant clinical trials identified.

**Figure A1:**
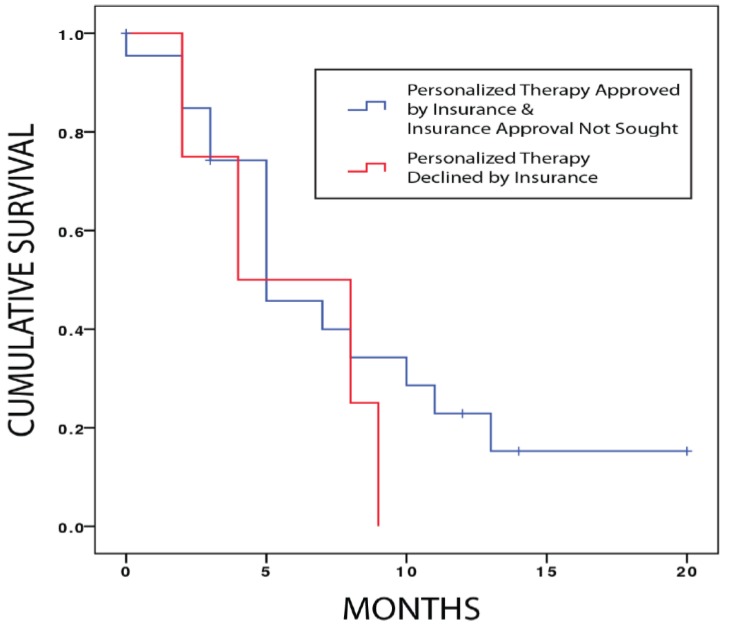
Progression-free survival of patients who had personalized therapy declined by insurance as compared to all other patients who underwent FoundationOne testing. Kaplan-Meier plot of progression-free survival in months for patients who applied for insurance reimbursement for personalized therapy based on FoundationOne result and had reimbursement declined (red line) as compared to all other patients who underwent FoundationOne testing (blue line). Progression-free survival was not significantly different between these groups (p = 0.334, Log Rank comparison). Cross-hatch lines represent data censored by the end of study period or when patient lost to follow up.

Among the 19 patients for whom a relevant FDA approved targeted therapy was identified by tumor profiling, six patients (31.5%) did not pursue a targeted therapy because they achieved an interval response to conventional treatment and three patients (15.7%) declined a targeted therapy when it was offered. A total of six patients sought insurance approval for a targeted therapy and two were declined (33% of those who sought approval). Median progression-free survival for patients who sought insurance approval for targeted therapy and were declined was 4 months (95% CI 0–9.8) and was not significantly different from the progression-free survival of 5 months (95% CI 2.3–7.7) for all other patients who underwent FoundationOne^TM^ testing (*p* = 0.334, Log Rank test) (Appendix Figure A1). Overall only one patient who underwent FoundationOne^TM^ tumor profiling for a gynecologic malignancy at our institution received a targeted therapy based on the tumor profile (1 of 26, 4%). This targeted therapy (ruxolitinib) was discontinued after a short period of treatment due to tumor progression, and the patient succumbed to her disease several months later. The most common targeted therapies suggested by FoundationOne^TM^ tumor profiling were everolimus/temsirolimus (suggested in 8/26 profiles, 30%) and trametinib (suggested in 8/26 profiles, 30%).

## 3. Discussion

For patients with gynecologic malignancies seen at our institution, treating clinicians most often sent tissue for commercial tumor profiling in the setting of recurrent serous epithelial ovarian, primary peritoneal, or fallopian tube carcinomas. Testing was typically performed after progression with multiple cytotoxic chemotherapy regimens. Regardless of tumor histology, the majority of patients who underwent tumor profiling did so in the setting of recurrent disease and nearly all patients had received some form of cytotoxic chemotherapy prior to obtaining tissue for tumor profiling. None of the patients in this series with ovarian, fallopian tube, or primary peritoneal cancer was a known germ line BRCA 1/2 mutation carrier, and therefore none would currently be a candidate for treatment with olaparib.

TP53 was the most frequently mutated oncogene identified by tumor profiling across all tumor types in this series. Among epithelial ovarian, primary peritoneal, or fallopian tube carcinomas, 9 of 12 tumor profiles identified TP53 mutations (75%). Thus TP53 mutations were somewhat less frequent in this series than in data from the Cancer Genome Atlas project, which identified TP53 mutations in 96% of samples from serous ovarian carcinomas [5]. Germ line RB1 mutations have been linked to an increased incidence of sporadic leiomyosarcoma, and it is interesting to note that all tumor profiles of leiomyosarcomas in this series identified RB1 mutations [9]. Three tumor profiles identified PTEN mutations and these were exclusively from the three endometrial that were submitted for testing.

It is notable that for 27% of all patients in this series (7/26), tumor profiling identified no mutations associated with a targeted therapy. This fraction is higher than the 18% advertised in product literature for this particular commercial tumor profile, a finding that may be partially explained by the fact that this series was confined to patients with gynecologic malignancies. When clinicians counsel patients on whether or not to send tissue for tumor profiling, it is therefore important that the discussion include the possibility that up to one in four tumor profiles may not reveal any mutations associated with targeted therapies.

Although the tumor profile suggested a potential clinical trial for 24 patients in this series (92%), significant barriers prevented patient access to these trials. For example, none of the clinical trials identified in any tumor profile were open for enrollment at our institution, an NCI-designated comprehensive cancer center and tertiary regional referral site. Geographic barriers to trial enrollment are not unique to our institution: of the 24 patients for whom a relevant clinical trial was identified in the tumor profile report, the nearest trial site for 7 patients was located in another state (29%). In addition, clinical trial eligibility of patients who underwent tumor profiling in this series may be limited by extensive exposure to prior courses of cytotoxic chemotherapy, with patients having undergone a median of two cytotoxic treatments prior to sample collection.

The list price of a tumor profile is $5800, although this cost may be reduced by various discounts in some circumstances. Based on this list price, at least $150,800 would be spent at our institution for each patient who received a targeted therapy. The vast majority of targeted therapies suggested by tumor profiles in this series were either kinase inhibitors (ruxolitinib, trametinib) or everolimus/temsirolimus, derivatives of sirolimus used in the treatment of renal cell carcinoma. The application of kinase inhibitors and everolimus/temsirolimus has been described in gynecologic malignancies, but the efficacy of these targeted therapies has not been validated in large randomized trials and none are FDA approved for use in these tumor types [10,11]. The lack of FDA approval may delay or restrict insurance approval for such therapies and it may therefore be reasonable for clinicians to seek insurance pre-approval for these medications prior to submitting a sample for profiling. If the cost of a targeted therapy cannot be borne by patient resources or insurance coverage, then the time and expense of tumor profiling could potentially be avoided.

Prospective phase II trials conducted under the NCI-MATCH schema will undoubtedly shed light on the clinical utility of tumor biomarker identification using next-generation sequencing [8]. Until these data are available, care must be taken to utilize tumor profiling in a manner that is cost-effective and minimizes unrealistic expectations in the ability of such testing to identify treatments that are both obtainable and effective.

## 4. Materials and Methods

The FoundationOne^TM^ test is a commercially available genomic profile generated using formalin-fixed paraffin embedded tissue obtained from either a primary solid tumor or a metastatic site [12]. A patient tissue sample is submitted to the company, where it is subjected to high throughput genetic sequencing of the entire coding sequence of 315 cancer-related genes in addition to the intronic regions of 28 additional genes. The sequencing results are analyzed and all identified mutations are then itemized and classified by mutation type (e.g., frame shifts, nonsense, deletion, *etc.*). Based upon the patient-specific mutation pattern, a tumor profile report is then generated that suggests relevant targeted therapies and clinical trials. At this time the FoundationOne^TM^ test is the most commonly used method for molecular profiling at our cancer center and all patients in this series had FoundationOne^TM^ testing. The decision to offer FoundationOne^TM^ testing was the prerogative of the treating oncologist. Data analysis consisted of descriptive statistics. The study protocol was approved by the IRB at our institution.

## 5. Conclusions

Targeted therapies will likely be a significant component of future treatment regimens for gynecologic malignancies. The commercial use of next-generation sequencing to generate personalized profiles of patient tumor samples has entered clinical practice and is being used to guide treatment decisions. However, commercial tumor profiling of gynecologic malignancies sometimes does not result in clinically actionable information, and significant geographic and logistical barriers prevent patient access to personalized treatment even when potential targeted therapies are identified.
